# Tunable Unexplored Luminescence in Waveguides Based on D-A-D Benzoselenadiazoles Nanofibers

**DOI:** 10.3390/nano14100822

**Published:** 2024-05-07

**Authors:** Carlos Tardío, Esther Pinilla-Peñalver, Beatriz Donoso, Iván Torres-Moya

**Affiliations:** 1 Department of Inorganic, Organic Chemistry and Biochemistry, Faculty of Chemical Science and Technologies, Instituto Regional de Investigación Científica Aplicada (IRICA), University of Castilla-La Mancha, 13071 Ciudad Real, Spain; carlos.tardio@uclm.es; 2 Department of Analytical Chemistry and Food Technology, University of Castilla-La Mancha, Avenue Camilo José Cela, s/n, 13071 Ciudad Real, Spain; esther.pinilla@uclm.es; 3 Department of Organic Chemistry, Faculty of Sciences, Campus of Fuentenueva, University of Granada, 18071 Granada, Spain; beatrizdonoso@ugr.es; 4 Department of Organic Chemistry, Faculty of Chemical Sciences, Campus of Espinardo, University of Murcia, 30010 Murcia, Spain

**Keywords:** benzoselenadiazoles, nanofibers, D-A-D systems, luminescence, waveguide

## Abstract

A set of novel Donor-Acceptor-Donor (D-A-D) benzoselenadiazole derivatives has been synthesized and crystallized in nanocrystals in order to explore the correlation between their chemical structure and the waveguided luminescent properties. The findings reveal that all crystals exhibit luminescence and active optical waveguiding, demonstrating the ability to adjust their luminescence within a broad spectral range of 550–700 nm depending on the donor group attached to the benzoselenadiazole core. Notably, a clear relationship exists between the HOMO-LUMO energy gaps of each compound and the color emission of the corresponding optical waveguides. These outcomes affirm the feasibility of modifying the color emission of organic waveguides through suitable chemical functionalization. Importantly, this study marks the first utilization of benzoseleniadiazole derivatives for such purposes, underscoring the originality of this research. In addition, the obtention of nanocrystals is a key tool for the implementation of miniaturized photonic devices.

## 1. Introduction

In the past decade, rapid advancements in the field of photonic devices have brought about an unprecedented transformation in optics, catalyzing breakthroughs spanning from communications to medicine [1,2,3,4]. In this dynamic landscape, optical waveguides have emerged as foundational building blocks, playing an irreplaceable role in manipulating and transmitting light at nanoscale dimensions. Their ability to guide light through microscopic structures has spearheaded a revolution in the miniaturization and efficiency of photonic systems [5,6,7,8].

Against the backdrop of this revolution, a pressing demand arises: the need for even smaller and more efficient photonic devices. Miniaturization has become a crucial pillar for developing more advanced technologies; in this context, nanoscale optical waveguides emerge as prominent players [9]. The capability of these structures to direct and modulate light at sub-microscopic levels proves crucial for adapting photonic devices to the emerging demands of the modern era. In addition, one of the main problems with organic crystals for implementation in photonic devices such as optical waveguides is their stiffness. In this sense, the search for flexible organic crystals has been a tremendously expanding line of research in recent years [10,11,12]. Obtaining nanostructures also contributes to the possibility of increasing their flexibility to be implemented in photonic devices, since longer structures are more difficult to deform and recover their original shape without breaking.

However, miniaturization alone is not sufficient. The precise modulation of emission color in these optical waveguides stands out as an imperative, radically transforming the functionality of these devices. From quantum communication to exploration in bio-photonics, the significance of accurately tuning emission color lies not only in miniaturization, but also in the ability to customize and optimize these structures at nanoscale levels [13].

In the last decade, the benzoselenodiazole core has emerged as a chemically intriguing entity, particularly in photophysical and electronic applications. It has exhibited notable electrochemical properties, propelling its application in electronic or photovoltaic devices [14,15,16]. This heterocyclic system, which amalgamates a benzene ring with a selenodiazole ring, has proven exceptionally promising in the design and development of compounds with unique optical and electronic properties. Additionally, this core possesses an acceptor character, easily modulating with the introduction of donor groups at positions 4 and 7, thereby forming donor-acceptor-donor (D-A-D) systems capable of modulating HOMO (Highest Occupied Molecular Orbital) and LUMO (Lowest Unoccupied Molecular Orbital) facilitating the generation of intramolecular charge transfer (ICT) states, resulting in significant bathochromic emission shifts and achieving red-shifting properties [17,18,19,20,21].

The strategic presence of selenium in the structure of benzoselenodiazole imparts distinctive photophysical characteristics to these compounds, making them ideal candidates for applications in optoelectronic devices. Their ability to absorb and emit light within specific ranges of the electromagnetic spectrum has spurred intensive research in the design of photosensitive materials and efficient light-emitting compounds [22,23]. However, its application in emissive optical waveguides is still unexplored, and to the best of our knowledge, there are no examples in the literature about this application, and this fact directed us to explore the employment of this moiety in this application.

**Figure 1:**
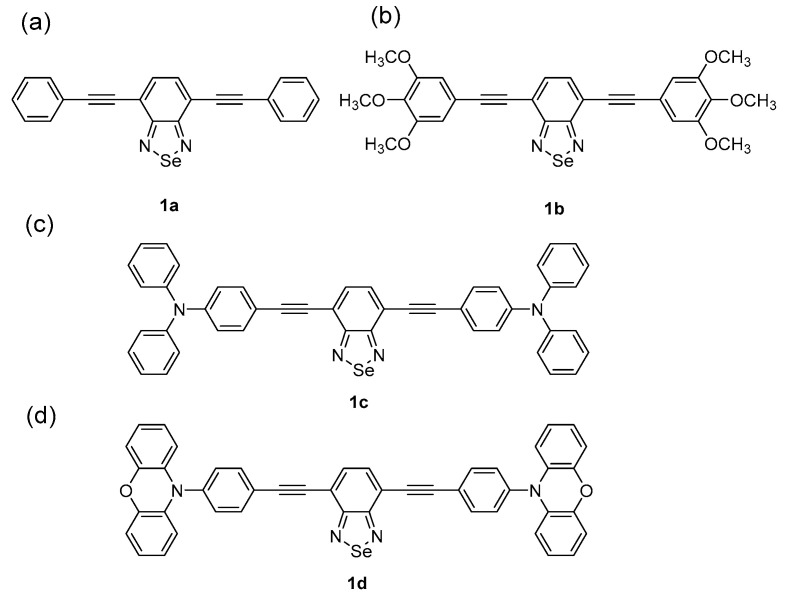
Chemical structures of the compounds **1a** (**a**), **1b** (**b**), **1c** (**c**) and **1d** (**d**), as described in this work.

Drawing from these characteristics and our prior exploration of benzoazole-based optical waveguides [24], different benzoselenadiazole derivatives were synthesized (**1**) with the introduction of four diverse donor groups in the peripheral positions 4 and 7, connected by a π-bridge through an alkynyl group, aiming to fine-tune the emission properties and build D-π-A-π-D architectures (Figure 1). Nanocrystalline forms of these compounds were cultivated via straightforward solution-based techniques for the investigation of their luminescent and light-conducting attributes in optical waveguides.

## 2. Materials and Methods

### 2.1. General Techniques

All the chemicals utilized in the experiments for the synthesis of derivatives **1** were procured commercially, except styrene, which underwent prior distillation. Reactions involving air-sensitive materials were conducted under an atmosphere of argon. Microwave irradiations were executed using a Discover^®^ (CEM, Matthews, NC, USA). focused microwave reactor. Silica gel, (Merck, Kieselgel 60, 230–240 mesh, Merck, Darmstadt, Germany), was employed for flash chromatography. Analytical thin layer chromatography (TLC) was carried out on aluminum-coated Merck Kieselgel 60 F254 plates (Merck, Darmstadt, Germany).

^1^H-NMR and ^13^C-NMR spectra collection were recorded using a Bruker Advance Neo NMR spectrometer that operates at 500.16 MHz for ^1^H and 125.75 MHz for ^13^C. All spectra were acquired at 298 K, employing partially deuterated solvents as internal references. Coupling constants (J) are expressed in hertz (Hz), and chemical shifts (δ) are reported in parts per million (ppm). Multiplicities are described in the following way: s = singlet, d = doublet, t = triplet, m = multiplet.

For elemental analysis experiments (EA) C, H, N and S micro-sample elemental analyzer LECO (model CNHS-932, St. Joseph, MI, USA) was used, employing 2 mg of sample for each experiment.

Mass spectra were obtained on a Bruker Autoflex II TOF/TOF (Bruker, Billerica, MA, USA) spectrometer using dithranol as the matrix for all the experiments.

UV–visible and fluorescence spectra studies in solution were carried out using a Jasco V-750 spectrophotometer (JASCO-Spain, Madrid, Spain) and Jasco FP-8300 spectrofluorimeter (JASCO-Spain, Madrid, Spain), respectively. The absorption and emission spectra were taken using dichloromethane as a solvent and at a concentration of 10^−5^ M at room temperature using standard quartz cells of 1 cm width and high spectroscopic grade solvents with very high purity.

Fluorescence images of the nanocrystals formed from derivatives **1** were captured using a Leica TCE SP2 (Wetzlar, Germany) confocal microscope equipped with a versatile mercury lamp capable of exciting fluorescence at various wavelengths. To achieve precise excitation and absorption, a set of filters was employed. These filters were designed to specifically target wavelengths within the blue spectrum (λexc = 320–380 nm, λem = 410–510 nm), green spectrum (λexc = 450–490 nm, λem = 515–565 nm) or red spectrum (λexc = 475–495 nm, λem = 520–570 nm).

### 2.2. Experimental Section

General procedure: A mixture of 4,7-dibromobenzo[c][1,2,5]selenadiazole (**2**) (0.100 g, 0.29 mmol), the corresponding acetylene derivative (**3**) (0.6 mmol), DBU (0.088 g, 0.58 mmol), CuI (0.003 g, 0.015 mmol) and Pd- EncatTM TPP30 (0.026 g, 0.01 mmol) was charged to a dried microwave vessel under an argon atmosphere. After that, 1 mL of CH_3_CN was added to the vessel, which was then closed and irradiated at 150 °C for 20 min in all the cases. The crudes’ reactions were purified by column chromatography using hexane/ethyl acetate as eluent to achieve analytically pure products **1**. It can be pointed out that for all the reactions, microwave irradiation was used as an energy source and Pd-EncatTM TPP30 was recovered after its use by filtration, and was used again for following reactions to reduce the environmental impact of the process. 

-4,7-bis(phenylethynyl)benzo[c][1,2,5]selenadiazole (**1a**): From ethynylbenzene (**3a**) (0.061 g, 0.6 mmol), derivative **1a** (0.095 g, 86%) was obtained as a yellow solid by column chromatography using hexane/ethyl acetate as eluent (9:1). ^1^H-NMR (CDCl_3_, 300 MHz), d: 7.54 (s, 2H, H_benzoselenazole_), 7.52–7.51 (m, 4H, H_phenyl_), 7.22–7.20 (m, 6H, H_phenyl_), ^13^C-NMR (CDCl_3_, 75 MHz), d: 155.5, 131.4, 129.6, 128.7, 128.5, 123.3, 110.0, 91.9, 75.6. MS (EI): *m*/*z*: 383.9 [M+]. EA; Calculated for C_22_H_12_N_2_Se; C: 68.94; H: 3.16; N: 7.31, found C: 68.97; H: 3.13; N: 7.28.

-4,7-bis((3,4,5-trimethoxyphenyl)ethynyl)benzo[c][1,2,5]selenadiazole (**1b**): From 5-ethynyl-1,2,3-trimethoxybenzene (**3b**) (0.115 g, 0.6 mmol), derivative **1b** (0.148 g, 91%) was obtained as a dark yellow solid by column chromatography using hexane/ethyl acetate (9:1) as eluent. ^1^H-NMR (CDCl_3_, 300 MHz), δ: 7.51 (s, 2H, H_benzoselenazole_), 6.86 (s, 4H, H_phenyl_), 3.82 (s, 18H, -OCH_3_), ^13^C-NMR (CDCl_3_, 75 MHz), d: 155.5, 154.8, 139.0, 129.6, 118.3, 110.0, 106.4, 95.0, 74.2, 60.7, 56.8. MS (EI): *m*/*z*: 565.41 [M+]. EA; Calculated for C_28_H_24_N_2_O_6_Se; C: 59.68; H: 4.29; N: 4.97, found C: 59.70; H: 4.32; N: 5.00.

-4,7-bis(diylbis(ethyne-2,1-diyl))bis(N,N-diphenylaniline)benzo[c][1,2,5]selenadiazole (**1c**) From 4-ethynyl-N,N-diphenylaniline (**3c**) (0.161 g, 0.6 mmol), derivative **1c** (0.162 g, 78%) was obtained as an orange solid by column chromatography using hexane/ethyl acetate (9:1) as eluent. ^1^H-NMR (CDCl_3_, 300 MHz), δ: 7.58 (s, 2H, H_benzoselenazole_), 7.49–7.47 (d, 4H, H_phenyl_), 7.26–7.23 (t, 8H, H_phenyl_), 7.09–7.07 (m, 12H, H_phenyl_), 6.98–6.94 (m, 4H, H_phenyl_). ^13^C-NMR (CDCl_3_, 75 MHz), d: 155.5, 145.6, 145.4, 131.4, 129.8, 129.6, 128.3, 126.3, 124.9, 119.7, 110.0, 91.9, 75.6. MS (EI): *m*/*z*: 719.1 [M+]. EA; Calculated for C_46_H_30_N_4_Se; C: 76.98, H: 4.21; N: 7.81, found C: 76.99, H: 4.22; N: 7.80.

-4,7-bis((4-(10H-phenoxazin-10-yl)phenyl)ethynyl)benzo[c][1,2,5]selenadiazole (**1d**). From 10-(4-ethynylphenyl)-10H-phenoxazine (**3d**) (0.170 g, 0.6 mmol), derivative **1d** (0.140 g, 65%) was obtained as a red solid by column chromatography using hexane/ethyl acetate (95:5) as eluent. ^1^H-NMR (CDCl_3_, 300 MHz), δ: 7.52–7.51 (d, 4H, H_phenyl_), 7.44 (s, 2H, H_benzoselenazole_), 7.32–7.30 (m, 4H, H_phenyl_), 7.24–7.18 (m, 12H, H_phenyl_), 7.16–7.15 (d, 4H, H_phenyl_). ^13^C-NMR (CDCl_3_, 75 MHz), δ: 155.5, 145.7, 144.9, 132.9, 131.5, 130.3, 129.6, 125.5, 125.2, 120.8, 119.4, 117.4, 110.0, 91.9, 75.6. MS (EI): *m*/*z*: 746.1 [M+]. EA; Calculated for C_46_H_26_N_4_O_2_Se; C: 74.09, H: 3.51; N: 7.51, O: 4.29, found C: 74.13, H: 3.49; N: 7.52, O: 4.29.

## 3. Results and Discussion

### 3.1. Synthesis

**Scheme 1:**
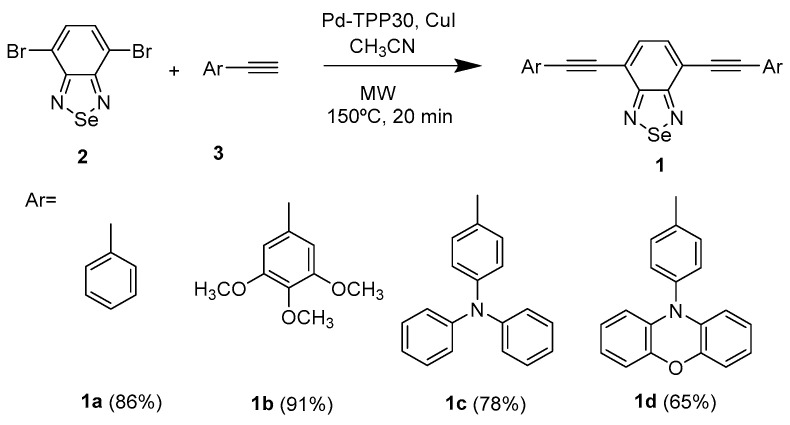
Synthesis of D-A-D benzoselenadiazoles derivatives **1**, as described in this work.

The synthesis of D-A-D benzoselenadiazole derivatives **1** was performed through a Sonogashira C-C cross-coupling reaction between derivatives **2** and **3** (Scheme 1), optimized previously by our research group for other azoles and benzoazoles [25,26]. All the reactions were carried out using microwave irradiation, with the reusable catalyst Pd-Encat TPP30 to increase the sustainability of the synthetic procedure, achieving the desired compounds **1** in 30 min in very good yields (65–91%), that gave adequate analytical NMR spectroscopic data (NMR spectra collection is recorded in Supporting Information section). We would like to point out that derivative **1a** had been previously synthesized by Li and co-workers under conventional conditions [27], improving the yield with this procedure using microwave irradiation as an energy source and considerably reducing the reaction time.

### 3.2. Theoretical Calculations

Before later optical characterization, the minimum-energy optimized structures were calculated at the B3LYP/6-31G (d,p) theoretical level [28,29] and are detailed in Table 1. Across all compounds, the HOMO molecular orbital is situated along the horizontal axis of the molecule, mainly affected by the donor group, while the LUMO orbital predominantly occupies the vertical axis, which mainly corresponds to the benzoselenadiazole core, existing as a shared overlap region, thus facilitating intramolecular charge transfer (ICT) [30].

The variation in donor peripheral groups (**1a**–**d**) correlates with the electron-donating tendency of the substituent, as indicated by the HOMO-LUMO gap. The most dramatical changes can be detected in HOMO values due to the changes in the donor group that implies changes in the HOMO-LUMO gaps, while LUMO values are not significantly affected because the major contribution is because the benzoselenadiazole moiety that keeps constant in all the derivatives. Specifically, compound **1a**, featuring peripheral phenyl groups (weaker donor groups), exhibits a higher HOMO-LUMO gap value compared to compounds **1b**–**d**. The introduction of methoxy groups in the periphery in **1b** with +I and +K characters increases the donor character and decreases the HOMO-LUMO gap with respect to **1a** (2.44 vs. 2.60 eV). Consequently, **1c**, with enhanced donor character due to its triphenylamine group, demonstrates a lower HOMO-LUMO gap value relative to **1a** and **1b** (2.20 vs. 2.60 and 2.44 eV). Finally, **1d**, with the strongest donor group, phenylphenoxazine, showed the lowest HOMO-LUMO gap (2.05 eV), and this fact will directly impact their photophysical properties.

### 3.3. Photophysical Studies

**Figure 2:**
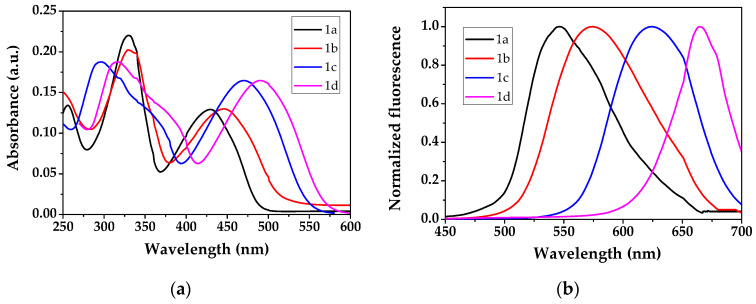
Absorption (**a**) and emission (**b**) spectra of derivatives **1** in 10^−5^ M CH_2_Cl_2_ solutions.

The UV-vis and fluorescence spectra of the different derivatives **1** were carried out in 10^−5^ M CH_2_Cl_2_ solutions. They are recorded in Figure 2 and photophysical data are summarized in Table 2.

It can be pointed out that the data recorded in Table 2 for **1a** agree with the data previously described by Li and co-workers for this compound [27]. The compounds **1a**–**d** exhibit absorption spectra characterized by a broad band with peaks ranging from around 300 to 330 nm, attributed to a π-π* transition, and a second band centered at longer wavelengths (around 430–490 nm), attributed to an ICT state. When the photoluminescence (PL) spectra are calculated following photoexcitation at the peaks of the longest-wavelength absorption band, the maxima shifts from 546 nm to 664 nm. This gradual red-shift of the PL spectra corresponds to the push–pull nature of the dyes, moving towards longer wavelengths as the donor-acceptor character strengthens. Consequently, **1d** displays the longest emission wavelength among all D-A-D derivatives due to the pronounced electron-donating properties of the phenylphenoxazine moiety. Similarly, **1c** also exhibits bathochromic shifts compared to **1a** and **1b**, attributed to the enhanced donor character of the triphenylamine derivative in comparison to phenyl or trimethoxyphenyl groups.

Consequently, the photoluminescence (PL) spectra of the derivatives **1** undergo spectral changes in alignment with the calculated HOMO-LUMO gap energies. The experimental optical HOMO-LUMO gaps were determined based on the onset of the lowest energy band absorption and demonstrated satisfactory agreement with theoretical predictions (Table 2). Additionally, it is noteworthy that a similar observation can be made regarding the spectral evolution of the lowest π-π* absorption transition..

Finally, the fluorescence quantum yields of these derivatives in solution were determined in CHCl_3_ using quinine sulfate in 0.1 M H_2_SO_4_ (Φ = 0.54) and fluorescein in 0.1 M NaOH (Φ = 0.79) for **1a**–**c**, and cresyl violet in ethanol (Φ = 0.56) for **1d** as standards, revealing moderate values (Table 2). The values of the quantum yield in the solid state (Table 2) are moderate and slightly decreased for all the derivatives in comparison with values in the solution state to values between 0.13 and 0.43.

### 3.4. Self-Assembling Studies

The creation of supramolecular assemblies using derivatives **1** was achieved through the slow diffusion method. In this procedure, a container holding a diluted solution (1 mg) of derivatives **1** in 1 mL of either chloroform or tetrahydrofuran, recognized as effective solvents, was carefully placed into another container containing a non-soluble poor solvent such as hexane, acetonitrile, ethanol or methanol. The second container was securely sealed and left undisturbed at room temperature. After a few days, the emergence of supramolecular aggregates becomes observable.

**Figure 3:**
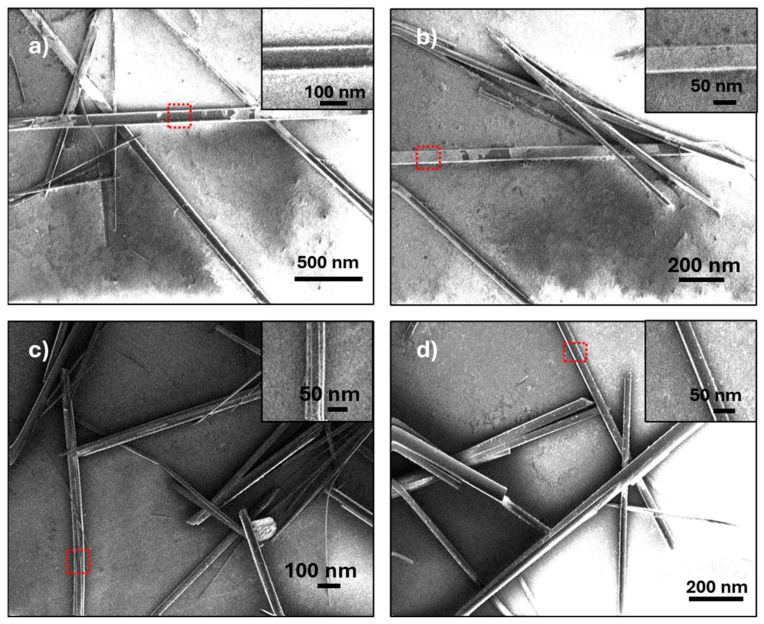
SEM images for the nanocrystals of (**a**) **1a** (THF/MeOH); (**b**) **1b** (CHCl_3_/EtOH); (**c**) **1c** (CHCl_3_/MeOH); (**d**) **1d** (THF/EtOH).

SEM images of the resultant nanofibers were acquired, with a focus on selecting the most promising ones in the form of fibers. Examples showcasing the optimal morphologies are presented in Figure 3, while additional SEM images can be found in the Supporting Information (Figure S1).

As we can observe in Figure 3, all nanocrystals exhibit a well-defined fibrillar shape, making them ideal candidates for investigating their optical waveguide properties. It can be pointed out that the best fibrillar morphologies were obtained using MeOH or EtOH as poor solvents.

### 3.5. Optical Waveguiding Behaviour

**Figure 4:**
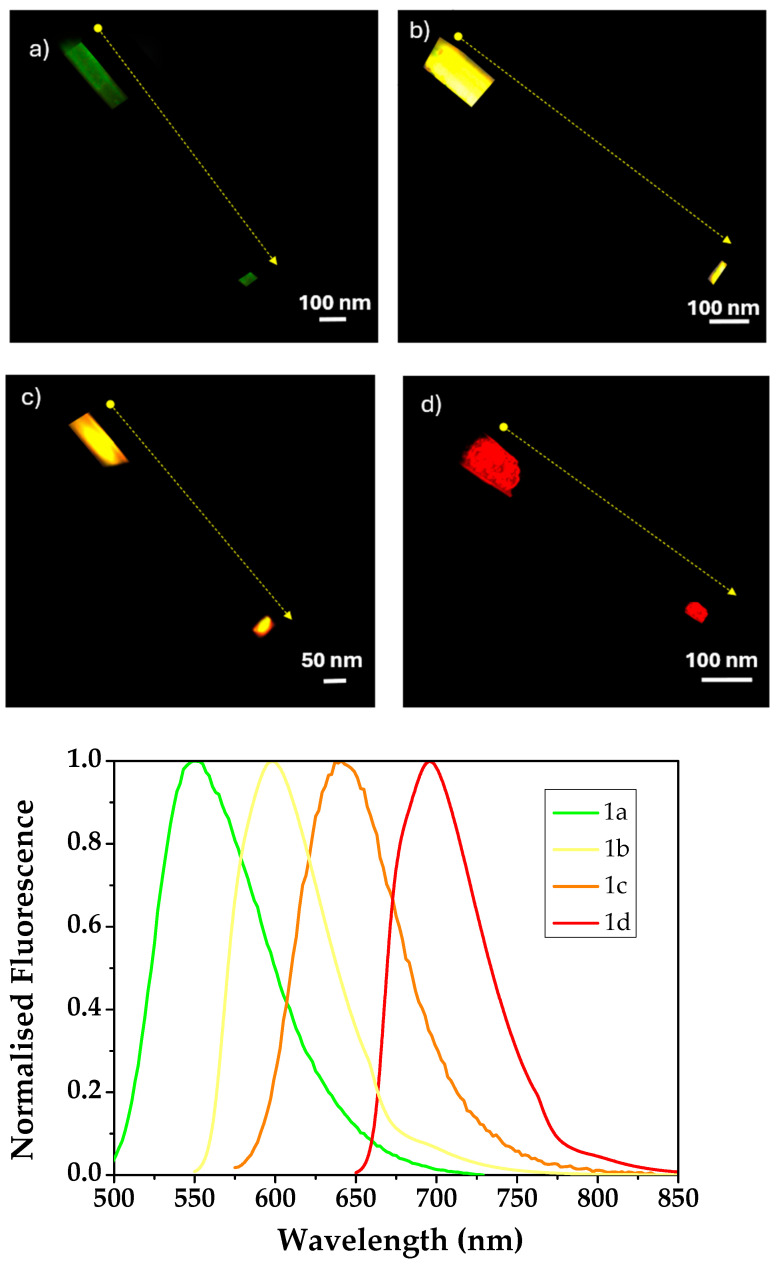
Confocal microscopy images and photoluminescence spectra for the nanocrystals of **1a** (**a**), **1b** (**b**), **1c** (**c**) and **1d** (**d**).

After identifying the most promising crystalline microfibers with suitable morphologies, confocal fluorescence microscopy images (Figure 4) of these nanocrystals were taken and recorded to assess their optical waveguiding characteristics.

**Figure 5:**
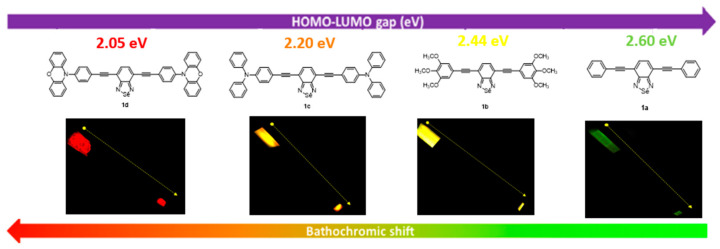
Theoretical HOMO-LUMO gap of derivatives **1a**–**d**/emission color relationship of the optical waveguides obtained from the nanocrystals of benzoselenadiazoles **1**.

The outcomes revealed that all nanocrystals of compounds **1a**–**d** exhibited luminescence. Upon irradiation with a 365 nm laser at the fiber body, it was notable that the emitted light propagated to the crystal’s tip, while the body itself brightened with emission. This phenomenon indicated strong luminescence with a distinct contrast between the bright tip/edges and the darker bulk, confirming efficient photon transport along the nanocrystals. Additionally, each nanocrystal was emitted at varying wavelengths, all distinct from the excitation wavelength, thus affirming their active optical waveguiding behavior (Figure 4). In addition, we can observe that in function on the molecular structure and the substitution in the benzoselenadiazole moiety, nanocrystals emitted a broad range of wavelengths, spanning from 550–700 nm. Notably, in the solid state, a significant redshift in PL is observed compared to the solution phase (Table 2 and Figure 2b vs. Figure 4). It is important to highlight that structural modifications of these derivatives exert an identical influence in both solution and solid states, resulting in consistent bathochromic shifts as the donor-acceptor (D-A) character of the derivative intensifies. Comparison of the emission wavelengths with theoretically calculated HOMO-LUMO gap values at the B3LYP/6-31G (d,p) theory level revealed a clear correlation, demonstrating the influence of different functional groups on the optical waveguide emission color. Fluorescence microscopy images revealed a progressive red-shift in the emission from green to red as theoretically calculated HOMO-LUMO gap energies decreased, indicating that TD-DFT could be utilized for on-demand emission tuning of the optical waveguides, and confirmed by the experimental HOMO-LUMO gaps obtained through the onset in absorption spectra. Like this, beginning with the highest HOMO-LUMO gap (2.60 eV), compound **1a** exhibited green emission (550 nm), compound **1b**, with the introduction of the trimethoxy groups, induces a lower HOMO-LUMO gap (2.44 eV), emitting in yellow (595 nm). The introduction of a stronger donor group like triphenylamine decreases the HOMO-LUMO gap (2.20 eV), achieving an orange color emission in the nanocrystals (640 nm). Finally, **1d**, with the strongest donor group (phenylphenoxazine), shows the lowest HOMO-LUMO gap (2.05 eV), with strong emission in red in the nanocrystals (698 nm). (Figure 5).

While numerous studies have explored this subject, as far as we know, there is currently no literature correlating the variation of emission color of a waveguide for benzoseleniadiazole derivatives, highlighting the novelty of this work.

**Figure 6:**
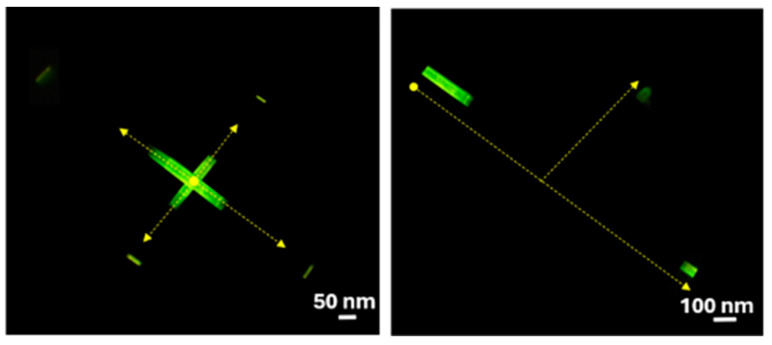
Confocal fluorescence microscopy images for interconnected fibers of **1a** showing the light transmission for different points in different directions.

Moreover, it is noteworthy that derivatives **1a**–**d** exhibit a tendency to form cross-linked and bunch-shaped nanocrystals as SEM images reveal. Irradiation of a single point on one fiber facilitated the light propagation in different directions; for example, in **1a** (Figure 6). This curious behavior, as described in recent literature [31,32,33], enhances the practical utility of these materials in real-world devices. It enables light to travel through different channels, facilitating parallel connectivity and interconnection between nanowires and various devices and facilitating the implementation in real miniaturized devices, which are very in demand these days in current society. For all these reasons, these results may be very useful in the coming years for the development of miniaturized photonic chips.

## 4. Conclusions

This study examines the impact of various peripheral donor groups incorporated into the benzoselenadiazole core, resulting in the design of diverse D-A-D derivatives. The investigation focuses on their performance as waveguides and their effect on emission color.

Nanocrystals formed via the self-assembly of the benzoselenadiazoles **1a**–**d** using the slow diffusion technique were examined through SEM images. The findings illustrate a significant inclination towards nanofiber formation, making them suitable for studies on optical waveguiding.

Each of the crystals of compounds **1a**–**d** exhibited efficient optical waveguiding behavior, accompanied by tunable color emissions ranging from green to red, contingent upon the nature of the peripheral donor groups. Notably, a clear correlation between the HOMO-LUMO gap and the emitted color of the optical waveguide exists. A reduced HOMO-LUMO gap resulted in a shift towards longer wavelengths in the emission spectrum. Compound **1d** shows the lowest HOMO-LUMO gap, and as a consequence, the most red-shifted emission.

This successful discovery could serve as a valuable approach for adjusting the emission color of optical waveguides, offering a systematic method for designing organic compounds and exploring their potential utilization as organic waveguides. Finally, the nanoscopic nature of these nanofibers and the properties described in this work constitute a promising study for the implementation of these nanofibers in real photonic devices, which are in high demand in current society.

## Figures and Tables

**Table 1:** Energies and topologies of the HOMO and LUMO frontier molecular orbitals, and HOMO-LUMO gaps of benzoselenadiazoles **1** obtained using DFT calculations at the B3LYP/6-31G (d,p) theory level.

Compound	HOMO (eV)	LUMO (eV)	HOMO LUMO Gap (eV)
**1a**	−5.66 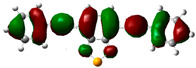	−3.06 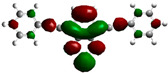	2.60
**1b**	−5.58 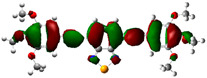	−3.14 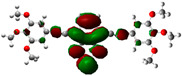	2.44
**1c**	−5.28 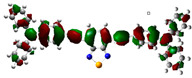	−3.08 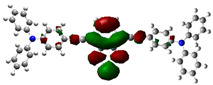	2.20
**1d**	−5.11 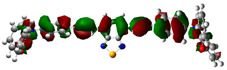	−3.06 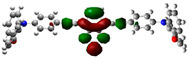	2.05

**Table 2:** Photophysical data of derivatives **1**.

Compound	λ_abs_ [a] (nm)	λ_em_ [b] (nm)	λ_emsol_ [c] (nm)	λ_onset_ [d] (nm)	Φ_dis_ [e]	Φ_sol_ [f]	HOMO-LUMO Gap [g] (eV)	HOMO-LUMO Gap [h] (eV)
**1a**	329, 434	546	550	488	0.20	0.13	2.60	2.54
**1b**	330, 448	574	595	514	0.54	0.43	2.44	2.41
**1c**	300, 470	625	640	552	0.69	0.41	2.20	2.25
**1d**	315, 491	664	698	585	0.60	0.35	2.05	2.11

[a] Absorption maxima of derivatives **1** in solution; [b] Emission maxima of derivatives **1** in solution; [c] Emission maxima of nanocrystals of derivatives **1**; [d] Onset wavelength calculated from the absorption spectra of the lowest energy band; [e] PL quantum yield in solution of derivatives **1**. [f] PL quantum yield measured in the solid state; [g] Theoretical HOMO-LUMO gap of compounds **1** computed at B3LYP/6-31G(d,p) level. [h] Experimental HOMO-LUMO gap of compound **1** calculated from the onset of the lowest energy absorption band using the 1240/λ_onset_ formula.

## Data Availability

Data are contained within the article.

## Supplementary Materials

The following supporting information can be downloaded at: https://www.mdpi.com/article/10.3390/nano14100822/s1, Figure S1: SEM images; Figures S2–S9: NMR spectra.
