# Supplementary material for: Tunable Unexplored Luminescence in Waveguides Based on D-A-D Benzoselenadiazoles Nanofibers
Source: Nanomaterials (Basel). 2024 May 7;14(10):822. doi: 10.3390/nano14100822 (PMC11124293; doi:10.3390/nano14100822)
Supplement: Supplementary file 1 [file nanomaterials-14-00822-s001.zip › nanomaterials-2968059-supplementary.pdf]

Supplementary Materials

Tunable Unexplored Luminescence in Waveguides Based on D-A-D Benzoselenadiazoles Nanofibers

Carlos Tardío [1], Esther Pinilla-Peñalver [2], Beatriz Donoso [3] and Iván Torres-Moya [4,*]

[1] Department of Inorganic, Organic Chemistry and Biochemistry, Faculty of Chemical Science and
Technologies, Instituto Regional de Investigación Científica Aplicada (IRICA), University of Castilla-La Mancha, 13071 Ciudad Real, Spain; carlos.tardio@uclm.es

[2] Department of Analytical Chemistry and Food Technology, University of Castilla-La Mancha, Avenue Camilo José Cela, s/n, 13071 Ciudad Real, Spain; esther.pinilla@uclm.es

[3] Department of Organic Chemistry, Faculty of Sciences, Campus of Fuentenueva, University of Granada, 18071 Granada, Spain; beatrizdonoso@ugr.es

[4] Department of Organic Chemistry, Faculty of Chemical Sciences, Campus of Espinardo, University of
Murcia, 30010 Murcia, Spain

* Correspondence: ivan.torres@um.es

Keywords: benzoselenadiazoles, nanofibers, D-A-D systems, luminescence, waveguide

1. SEM Images

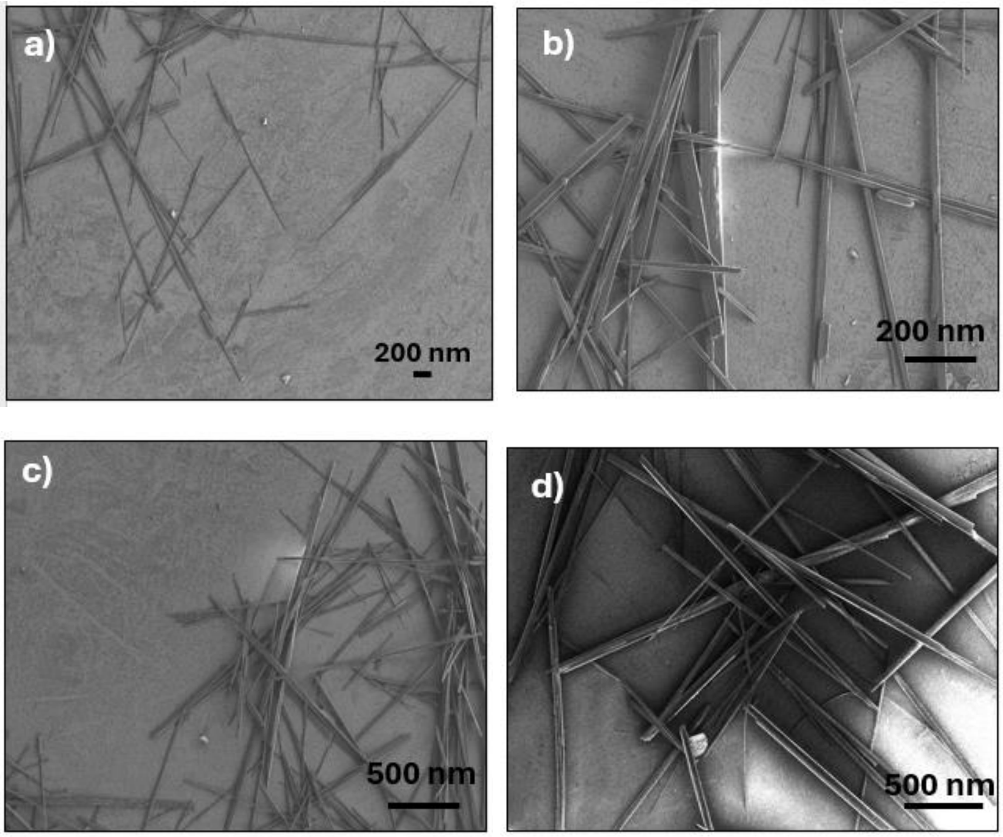

Figure S1. SEM images for the crystals of a) **1a** ($CHCl_3$/EtOH); b) **1b** (THF/Ethanol); c) **1c** (THF/MeOH); d) **1d** ($CHCl_3$/MeOH).

2. NMR spectra

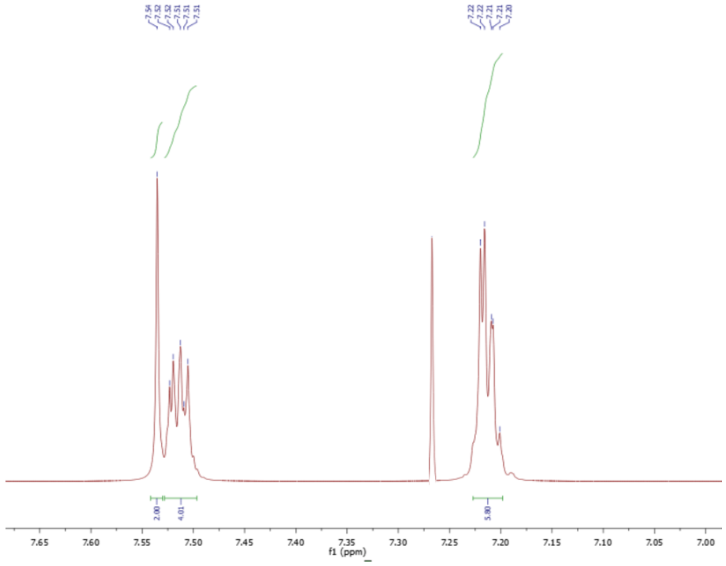

Figure S2. ^{1}H-NMR spectrum of **1a**

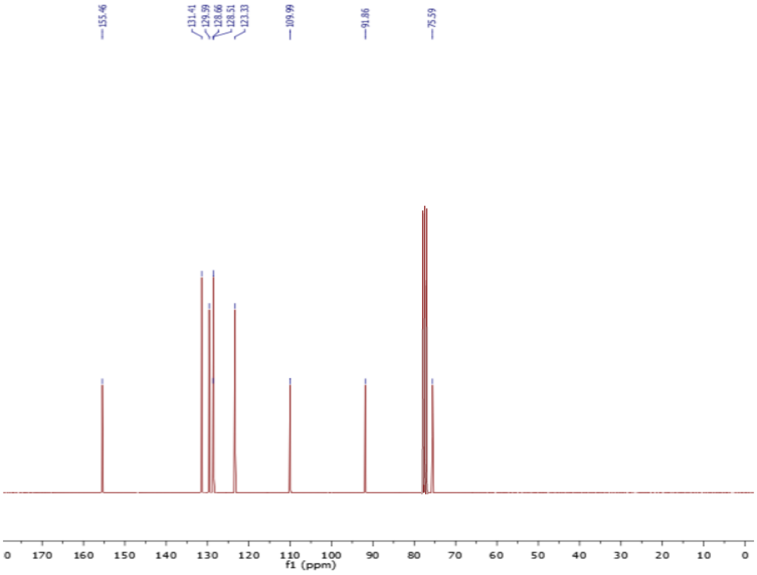

Figure S3. ^{13}C-NMR spectrum of **1a**

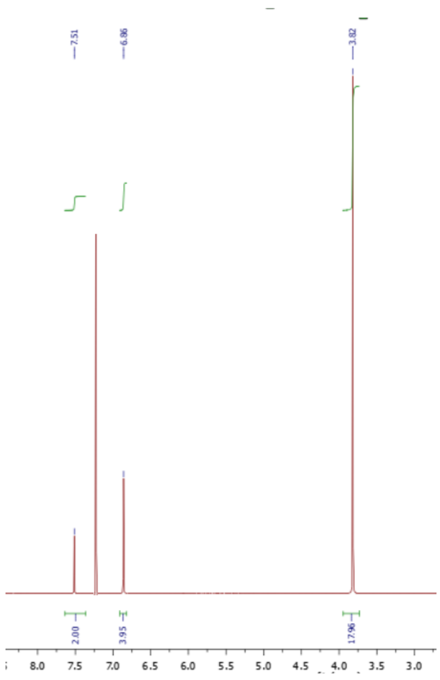

Figure S4. ^{1}H-NMR spectrum of **1b**

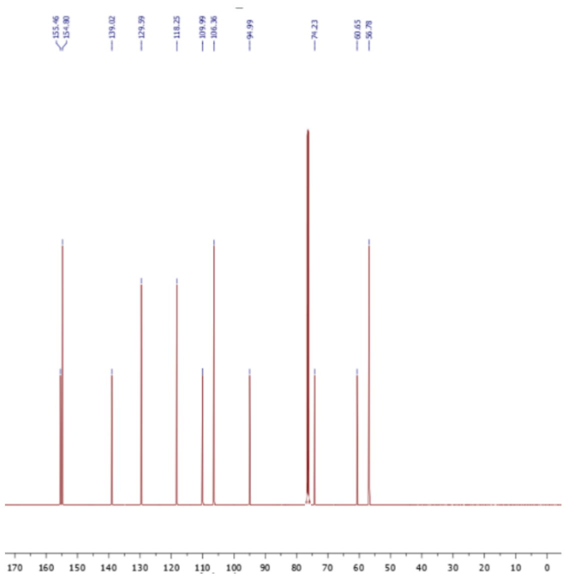

Figure S5. ^{13}C-NMR spectrum of **1b**

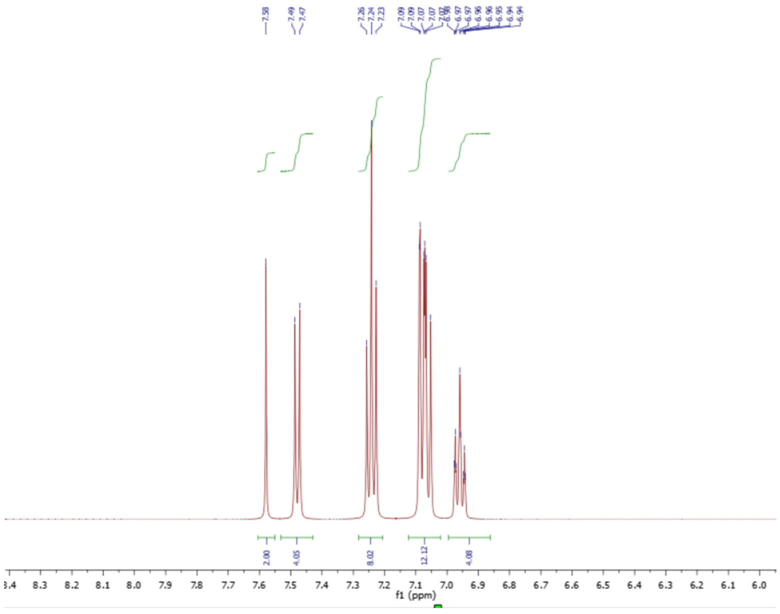

Figure S6. ^{1}H-NMR spectrum of **1c**

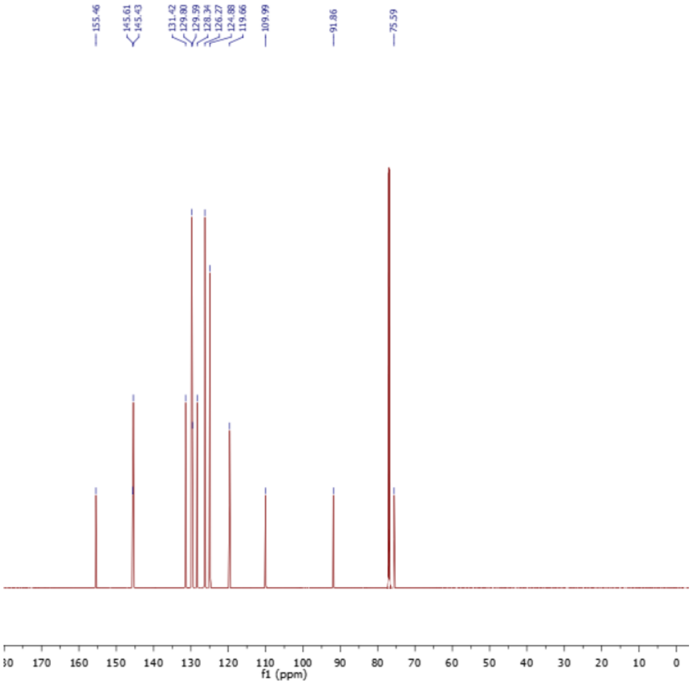

Figure S7. ^{13}C-NMR spectrum of **1c**

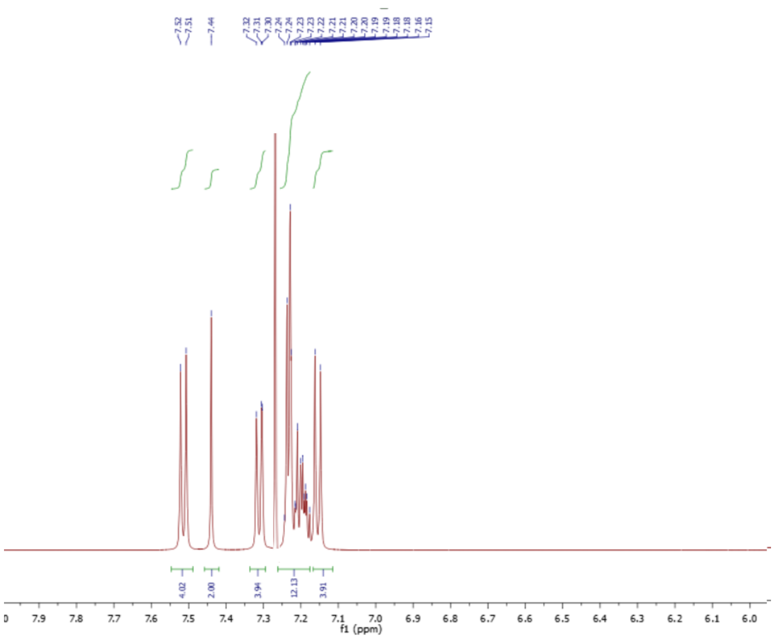

Figure S8. ^{1}H-NMR spectrum of **1d**

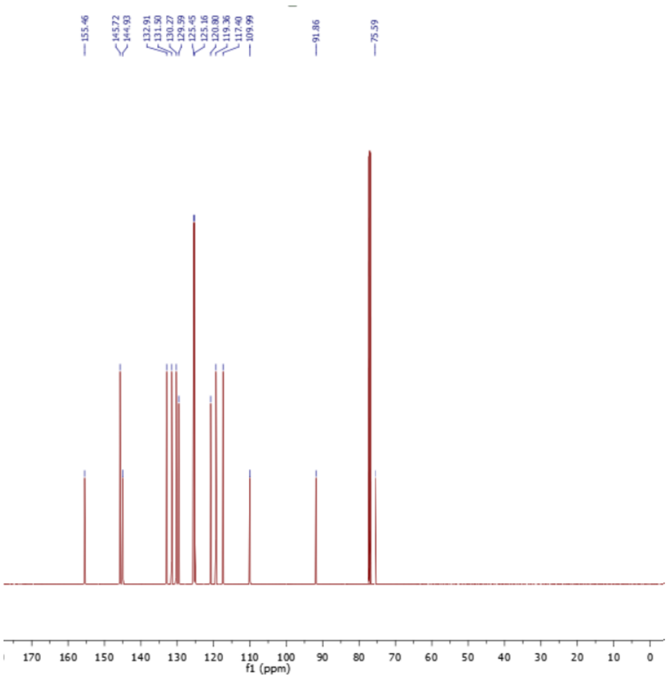

Figure S9. ^{13}C-NMR spectrum of **1d**
